# Outcomes with radiotherapy in multimodality treatment for hepatocellular carcinoma with portal vein tumour thrombosis

**DOI:** 10.1093/bjro/tzaf002

**Published:** 2025-02-18

**Authors:** Puja Sahai, Hanuman Prasad Yadav, Ashok Choudhury, Saggere Muralikrishna Shasthry, Ankur Jindal, Aprajita Mall, Amar Mukund, Yashwant Patidar, Mangu Srinivas Bharadwaj, Bangkim Chandra Khangembam, Guresh Kumar, Archana Rastogi, Viniyendra Pamecha

**Affiliations:** Department of Radiation Oncology, Institute of Liver and Biliary Sciences, New Delhi 110070, India; Department of Radiation Oncology, Institute of Liver and Biliary Sciences, New Delhi 110070, India; Department of Hepatology, Institute of Liver and Biliary Sciences, New Delhi 110070, India; Department of Hepatology, Institute of Liver and Biliary Sciences, New Delhi 110070, India; Department of Hepatology, Institute of Liver and Biliary Sciences, New Delhi 110070, India; Department of Radiation Oncology, Institute of Liver and Biliary Sciences, New Delhi 110070, India; Department of Interventional Radiology, Institute of Liver and Biliary Sciences, New Delhi 110070, India; Department of Interventional Radiology, Institute of Liver and Biliary Sciences, New Delhi 110070, India; Department of Nuclear Medicine, Institute of Liver and Biliary Sciences, New Delhi 110070, India; Department of Nuclear Medicine, Institute of Liver and Biliary Sciences, New Delhi 110070, India; Department of Biostatistics, Institute of Liver and Biliary Sciences, New Delhi 110070, India; Department of Pathology, Institute of Liver and Biliary Sciences, New Delhi 110070, India; Department of Hepatopancreatobiliary and Liver Transplant Surgery, Institute of Liver and Biliary Sciences, New Delhi 110070, India

**Keywords:** hepatocellular carcinoma, thrombosis, radiotherapy, tyrosine kinase inhibitors, immune checkpoint inhibitors, therapeutic chemoembolization

## Abstract

**Objectives:**

The purpose of the present study was to evaluate outcomes with radiation therapy (RT) in multimodality treatment for inoperable hepatocellular carcinoma (HCC) with portal vein tumour thrombosis (PVTT).

**Methods:**

The present retrospective study included 24 patients without extrahepatic metastases. The patients had received drug eluting beads - transarterial chemoembolization (DEB-TACE) (*n* = 10) and systemic treatment (*n* = 14) before RT. The dose fractionation was 12–31.5 Gy in 3–7 fractions of 4–5 Gy to PVTT or PVTT plus the liver parenchymal tumour. All patients were advised systemic treatment with sorafenib, lenvatinib, or nivolumab after RT. After RT, patients had received DEB-TACE within 8 weeks (*n* = 2) or at 5–10 months (*n* = 3). Treatment response was evaluated as per mRECIST and PERCIST, and Kaplan-Meier survival analysis was performed.

**Results:**

The disease control rate in PVTT was 50% at 3 months. The median overall survival (OS) was 10.9 months (95% CI, 0.74-21) for all patients. The 6-month, 1-year, 2-year, and 3-year OS rates were 75%, 45.8%, 25%, and 12.5%, respectively. The median OS was 30.4 months (95% CI, 12.1-48.7) versus 18.1 months (0.00-38.8) with complete or partial response versus stable or progressive disease in PVTT (*P* = .036). Eleven patients had a decline in Child Pugh score of 2 or more points within 3 months after RT. One patient underwent live donor liver transplantation (LDLT) and complete necrosis with no viable tumour was observed in the explant. The patient is cancer- and liver disease-free at 1 year after LDLT.

**Conclusions:**

The present study showed the benefit of radiotherapy with systemic therapy and DEB-TACE in patients with HCC with PVTT.

**Advances in knowledge:**

Radiotherapy as part of the multimodality treatment offers the potential to improve disease control and survival in patients with HCC with PVTT.

## Introduction

Liver cancer is the sixth leading site and the third common cause of mortality among all cancers as per the GLOBOCAN reports.^1^ Patients with hepatocellular carcinoma (HCC) commonly manifest with portal vein tumour thrombosis (PVTT) resulting in advanced disease presentation. Patients with PVTT are usually treated with systemic therapy as per the recommended guidelines. However, there are challenges to treat patients with vascular invasion because of the underlying chronic liver disease (CLD). In view of the vascular involvement, distant metastases are often observed at diagnosis or during the course of the illness. Therefore, multimodality therapeutic approaches are needed for an effective disease control to improve outcomes in patients with HCC with PVTT.

Patients with PVTT need combination treatments with resection and downstaging therapies are needed prior to liver transplantation. The survival in untreated HCC with PVTT ranges from 2 to 4 months. Locoregional treatments, that is, transarterial chemoembolization (TACE), selective internal radiation therapy (SIRT), microwave ablation (MWA), and/or radiotherapy for the parenchymal tumour and/or PVTT are increasingly being evaluated in combination with systemic therapy for improving disease control.^2^ Furthermore, a selected group of patients with liver limited disease can be offered definitive treatment options for a long-term survival.

Radiation therapy is a non-invasive modality with a special role in treating PVTT. The modern radiation techniques with hypofractionation create a sharp dose gradient resulting in a high dose to the tumour with an efficient sparing of surrounding organs at risk. Radiotherapy is given with the intent of reducing tumour load in advanced HCC and to make the patients respond better to systemic therapy, that is, tyrosine kinase inhibitors (TKIs) and/or immunotherapy.^3^^,^^4^ The addition of TACE further adds to the tumour response. The effect of combined modalities contributes towards reducing the tumour burden in HCC. The purpose of the present study was to evaluate outcomes with radiotherapy in multimodality treatment for patients with HCC with PVTT.

## Methods

### Eligibility criteria and work-up

We identified and retrospectively reviewed 24 patients with newly diagnosed inoperable HCC with PVTT without extrahepatic metastases and treated with RT with systemic therapy and TACE from November 2018 to January 2021. The patients were discussed in our multidisciplinary tumour board for the disease management. All patients had signed the informed consent forms for the procedures and treatments.

The presenting disease symptoms varied from abdominal pain, abdominal heaviness, abdominal distension, generalized fatigability, fever, and/or jaundice at the time of diagnosis of cancer. The patients underwent blood investigations with complete blood count (CBC), kidney function tests, liver function tests (LFT), and prothrombin time–international normalized ratio (PT-INR). Tumour markers, that is, alpha-fetoprotein (AFP) and protein induced by vitamin K absence-II (PIVKA-II) were tested in serum samples of patients.

The patient and disease characteristics are listed in Table 1. The patients’ ECOG performance status was 1 (*n* = 22) or 2 (*n* = 2). All patients had a haemoglobin level of ≥8 gm/dL. All patients had a PT-INR of ≤1.7. Thirteen patients had serum AFP more than 400 ng/mL at diagnosis. Three patients had normal serum AFP with raised PIVKA-II at diagnosis. The serum PIVKA-II level was raised in 23 patients at diagnosis. The remaining 1 patient did not have serum PIVKA-II level available. The patients were diagnosed with HCC on the multiphasic imaging as per the AASLD guidelines.^5^^,^^6^ Two patients had microscopic diagnosis as well with biopsy and cytology with cell block, respectively. In addition to the multiphase contrast-enhanced CT (CECT) abdomen, a whole body 18Fluorine fluorodeoxyglucose PET–contrast-enhanced CT (18F FDG PET-CECT) was performed in 17 patients for disease characterization, staging, and response assessment. The PVTT was diagnosed on the basis of arterial phase enhancement and venous washout in a filling defect with or without FDG uptake. The extent of PVTT was classified as proposed by the Liver Cancer Study Group of Japan. In 3 patients, the vp4 thrombus was extending further up to the splenoportal confluence (*n* = 1), splenoportal confluence, superior mesenteric vein, and splenic vein (*n* = 1), or paraumbilical vein (*n* = 1). The patients with nodal or distant metastases were excluded from the present study. In the 17 patients who had undergone 18F-FDG PET-CECT, a median SULpeak of 5.6 (range, 2.4-10.6) was noted in the liver parenchymal lesion. The median SULpeak was 5.3 (range, 2.8-12.2) in the PVTT in 15 patients. In the remaining 2 patients, the PVTT was non FDG-avid while the parenchymal lesion showed FDG avidity. In 16 patients, the median tumour size of the largest lesion was 7.75 cm (range, 2.8-15.9 cm). In the remaining 8 patients, the lesion(s) were ill-defined and infiltrative. Of the 16 patients, 15 had tumour size greater than 5 cm. The number of intrahepatic parenchymal lesions were one (*n* = 18), two (*n* = 1), or multiple (*n* = 5). Twenty-two out of the 24 patients had serum albumin >2.8 g/dL at diagnosis.

**Table 1.** Patient and disease characteristics.

Characteristic	Number
Patients	24
Age (years)	Median 57
	Range 33-73
Gender	
Male	24
Female	0
BCLC classification	
C	24
Cause of cirrhosis	
Non-alcoholic steatohepatitis	11
Hepatitis B virus	9
Hepatitis C virus	2
Ethanol	1
Hepatitis C virus + ethanol	1
Platelet count at diagnosis (per microlitre)	
150 000 and above	11
100 000–150 000	4
50 000–100 000	9
Serum bilirubin at diagnosis (mg/dL)	Range 0.5-4.1
>3	2
Serum albumin at diagnosis (g/dL)	Range 2.3-4.38
≥3.5	9
<3.5	15
ALBI grade at diagnosis	
1	2
2	19
3	3
ALBI grade before RT	
1	4
2	16
3	4
Child Pugh class and score at diagnosis	
A5	6
A6	6
B7	9
B8	2
B9	1
Child Pugh class and score before RT	
A5	6
A6	7
B7	10
B8	1
Type of portal vein tumour thrombosis	
vp1	0
vp2	4
vp3	6
vp4	14
Serum alpha-fetoprotein at diagnosis (ng/mL)	Median 631.6
	Range 8.12-72147.2
Serum PIVKA-II at diagnosis (mAU/mL)	Median 4870.9
	Range 408-100171.9
Liver tumour size maximum dimension (cm)	Median 7.75
	Range 2.28-15.9

Abbreviations: RT = radiation therapy; ULN = upper limit of normal.

Normal range: Platelet count = 150 000-450 000 per microlitre, serum albumin = 3.5-5.2 g/dL, serum total bilirubin = 0.2-1.1 mg/dL, serum direct bilirubin = 0.11-0.42 mg/dL, serum indirect bilirubin = 0.2-0.8 mg/dL, serum aspartate transaminase = 5-40 IU/L, serum alanine transaminase ≤30 IU/L, serum alkaline phosphatase = 30-120 IU/L, serum gamma glutamyl transpeptidase = ≤55 IU/L, serum alfafetoprotein = 0-8.5 ng/mL, and serum PIVKA-II = 1-40 mAU/mL.

The patients were worked-up for CLD with upper gastrointestinal endoscopy and had received appropriate management along with the cancer-directed therapies. Human albumin infusions were administered intravenously in patients with low serum albumin (<3.5 g/dL) before and during the course of liver-directed treatments and systemic therapy. The patients with mild ascites were treated with RT as and when there was resolution of ascites after receiving supportive treatment.

### Treatment before radiotherapy

The treatment details are mentioned in Table 2. All patients had received a course of intravenous high-dose vitamin K. The patients had received drug eluting beads - transarterial chemoembolization (DEB-TACE) with doxorubicin (*n* = 10) with 4 of them treated with sorafenib and 2 with lenvatinib before RT. The number of sessions of DEB-TACE ranged from 1 to 3. MWA was performed with DEB-TACE in 1 out of the 10 patients. The patient treated with SIRT had received sorafenib as well. One patient had received MWA prior to RT. Systemic treatment was given before RT in a total of 14 patients. Seven out of the 14 patients had received systemic treatment with sorafenib (*n* = 2), lenvatinib (*n* = 1), or nivolumab (*n* = 4) before RT. Out of the 4 patients, 2 had received 7 doses of nivolumab while 2 had received 1 dose of nivolumab. Sorafenib was given at a dose of 400 mg or 200 mg BD depending upon the patient’s tolerance and liver function. Lenvatinib was given at a dose of 8-12 mg OD. Dose was modified as per the patient’s tolerance and LFT values. Nivolumab was given at a dose of 3 mg/Kg intravenously every 2 weeks.

**Table 2.** Treatment details of patients with hepatocellular carcinoma with portal vein tumour thrombosis.

Characteristic	Number
Locoregional treatment before RT	
DEB-TACE	9
DEB-TACE with MWA	1
MWA	1
SIRT	1
Systemic treatment before RT	14
Site of RT	
PVTT +liver parenchymal tumour	14
PVTT	10
GTV (cm^3^)	Median 278
	Range 86.4-1697.4
PTV (cm^3^)	Median 673.8
	Range 302.4-2808.7
PTV CI	0.94-0.99
PTV HI	1.1-1.38
PTV V100% (%)	95-99.9
PTV D0.03 cm^3^ (%)	Median 130
	Range 117.5-167
PTV mean dose (Gy)	Median 33.3 Gy
	Range 14.3-41.5
Liver total volume (cm^3^)	Median 1711.4
	Range 848-2763.2
Liver minus ITV (cm^3^)	Median 1086.3
	Range 426.6-2378.1
Liver minus ITV Dmean (Gy)	Median 13.3
	Range 3.97-19.6
Liver-directed treatment after RT	
DEB-TACE	5
LDLT	1
Systemic treatment after RT	
Sorafenib	7
Sorafenib followed by lenvatinib	3
Lenvatinib	2
Nivolumab	3
Nivolumab and lenvatinib	4
Not given	5

Abbreviations: CI = conformity index; DEB-TACE = drug eluting beads - transarterial chemoembolization; GTV = gross tumour volume; HI = homogeneity index; ITV = internal target volume; LDLT = live donor liver transplantation; MWA = microwave ablation; PTV = planning target volume; PVTT = portal vein tumour thrombosis; RT = radiation therapy; SIRT = selective internal radiation therapy.

### Radiotherapy

Radiotherapy was started at a median of 9.29 weeks (range, 1-36 weeks) from the diagnosis. A planning multiphasic CECT with plain, arterial, late arterial, portovenous, and venous phases was performed with the patient positioned on a customized vacloc cushion bag for immobilization (GE Discovery^TM^ 710 128 slice PET/CT cum 4DCT simulator). The intravenous contrast agent, that is, omnipaque or visipaque was injected at a dose of 1.5 mL/kg at a rate of 3.5 to 4 mL/s with bolus tracking timing. The scan was performed in free breathing or with abdominal compression as per the patient’s comfort. The slice thickness was 2.5 mm. Additionally, a 4-D CT was performed using the Anzai respiratory system belt (AZ-733VI), Tokyo, Japan.

On the CT simulation scan, gross tumour volume (GTV) was delineated on the portovenous, or late arterial, or venous or the average dataset of 4-DCT. The GTV included both the PVTT and the liver parenchymal tumour (*n* = 14) or only the PVTT (*n* = 10) depending upon the volume of liver available for sparing. Among the 10 patients, the adjacent MWA cavity was included in the target volume with PVTT in 1 patient. The target volumes were drawn so as to spare ≥700 cm^3^ of liver. Five patients had multiple intrahepatic lesions. In 4 of the 5 patients with multiple lesions, the GTV included the PVTT plus the adjacent largest liver parenchymal lesion while only the PVTT in the remaining 1 patient. The GTV_primary and GTV_PVTT were separately drawn and different doses were prescribed to both in 3 patients. The internal target volume (ITV) margins were estimated from the 10 phases of 4-D CT as per the movement noted on the average dataset. The planning target volume (PTV) was generated as an expansion of 5 mm on the ITV. The dose limiting organs at risk (OARs), that is, liver, oesophagus, stomach, duodenum, bowel, heart, skin, chest wall, etc were delineated. A 1 cm margin for planning risk volume (PRV) was created on the gastrointestinal luminal organs at risk.

The dose fractionation was prescribed as 12-31.5 Gy in 3-7 fractions of 4-5 Gy each given as daily fractions with 5 in a week in the majority of patients. As most of the patients, *n* = 18 (75%) had vp3 or vp4 PVTT, the dose per fraction was kept at 4-5 Gy to decrease the risk of central hepatobiliary and gastrointestinal toxicity. The dose per fraction was chosen based on the volume of overlap of PTV with stomach, duodenum, large bowel, heart, etc. The dose fractionation regimes given were as follows: 30 Gy in 6 fractions with 5 Gy per fraction (*n* = 11), 31.5 Gy in 7 fractions with 4.5 Gy per fraction (*n* = 5), 27 Gy in 6 fractions with 4.5 Gy per fraction (*n* = 1), 12-28 Gy in 3-7 fractions with 4 Gy per fraction (*n* = 3). The dose of 35 Gy in 7 fractions was prescribed in 1 patient and 32 Gy in 8 fractions in 2 patients. One patient was given 30 Gy in 5 fractions with 6 Gy every alternate day fraction. Two out of the 24 patients were prescribed 20.5 and 31.5 Gy in 7 fractions and 28 and 30 Gy in 6 fractions to the liver primary parenchymal lesion and PVTT, respectively. The plans were generated with volumetric modulated arc technique (VMAT) calculated on the Monaco Treatment Planning System software (version 5.51). The prescription dose covered ≥95% of the PTV and ≥99% of the ITV. The stereotactic technique was used to create a dose fall-off at a ring between 2 and 3 cm around the PTV for sparing of uninvolved liver and gastrointestinal structures. The maximum point dose to 0.03 cm^3^ of PTV ranged from 111% to 167%. The volume receiving >105% dose outside the PTV was limited to <15% of the PTV volume. The 50% prescription isodose was aimed for fall-off at 2-3 cm around the PTV.

The dose volume constraint to liver minus ITV for prescription of 5 Gy per fraction was given as preferably mean dose ≤15 Gy or ≤10 Gy for patients with Child Pugh (CP) class A or B, respectively. Additionally, the volume of liver minus ITV receiving ≤15 Gy and ≤10 Gy was aimed at ≥700 cm^3^ for CP A and B, respectively. In the patients receiving 4-4.5 Gy per fraction, the liver minus ITV mean dose was allowed up to ≤24 Gy and ≤20 Gy for CP A and B, respectively. Two out of the 24 patients had volume of liver minus ITV of <700 cm^3^ (644.6 and 426.6 cm^3^). The patients with 644.6 and 426.6 cm^3^ volumes had mean dose in liver minus ITV of 13.5 Gy and 3.97 Gy, respectively. The maximum point dose constraint to 0.03 cm^3^ volume of the gastrointestinal luminal organs, that is, oesophagus, stomach, duodenum, small bowel, and large bowel was maintained at EQD2 of 54 Gy at α/β of 3. Radiation arcs were planned to avoid the beam entry through the uninvolved lobe of the liver. Incidentally, the intrahepatic lesions not covered in the target received the low dose fall-off irradiation. The patients were treated with RT on the Versa HD linear accelerator (Elekta Oncology Systems, Crawley, UK) with cone-beam CT (CBCT) image guidance for each fraction with matching of both soft tissue and bone. The patients were monitored with complete blood counts and liver function tests during RT. Radiotherapy was interrupted if serum bilirubin reached more than 3 mg/dL. The patients were given intravenous or oral antiemetic and proton pump inhibitor or antacid medications during the course of RT. Proton pump inhibitor or antacid medication was continued orally for a duration of 4-6 weeks after RT. Human albumin infusions were given to the patients as needed.

### Systemic therapy

All patients were advised systemic treatment with sorafenib, lenvatinib, or nivolumab after RT. Systemic therapy was started within 1-2 weeks after the last fraction of RT after clinical evaluation and checking of blood parameters with CBC, LFT, KFT, PT-INR. The starting dose of sorafenib was 200 mg OD or BD and was maintained at 200 mg BD as per the patient’s tolerance and LFT values. The starting dose of lenvatinib was 4 or 8 mg per day after RT. The patients were monitored with clinical examination and blood investigations with CBC, KFT, LFT, and PT-INR. The dose of sorafenib or lenvatinib was modified as per the patient’s tolerance and LFT values. Sorafenib or lenvatinib was discontinued at serum bilirubin more than 3 mg/dL. Nivolumab was given at a dose of 3 mg/Kg intravenously every 2 weeks for 6-20 doses. Human albumin infusions were administered intravenously as needed.

### Assessment and follow-up

Treatment response was assessed with blood investigations and tumour markers and multiphasic CECT abdomen at 4-6 weeks after the last fraction of RT. A multiphasic CECT abdomen with whole body 18F-FDG PET-CT was performed at 12 and 24 weeks after RT. Thereafter, the patients were followed-up every 3 months with blood investigations, tumour markers, and imaging as needed. The response was assessed in terms of decrease in arterial enhancement and FDG uptake with or without shrinkage of tumour and thrombus. The response assessment was documented as per the mRECIST^7^ and PERCIST^8^ criteria. The best response after RT was documented. The objective response rate (ORR) was defined as the percentage of patients with complete response (CR) or partial response (PR). The disease control rate (DCR) was defined as the percentage of patients with CR, PR, or stable disease (SD). Locoregional treatment after RT was given as needed based on the assessment with tumour markers and imaging. Two patients had received DEB-TACE within 8 weeks after RT. Another 3 patients had received DEB-TACE at 5 months (*n* = 1) or 10 months (*n* = 2) after RT. The systemic therapy drug was changed as per the patient’s tolerance and response on follow-up. Toxicity was graded as per the Common Terminology Criteria for Adverse Events (CTCAE) version 5.0. The changes in CP score were noted at follow-up after RT. The data cut-off for the last follow-up was on 31 December 2023. The primary end points were response and overall survival (OS). The secondary end point was toxicity.

### Statistical analysis

Descriptive statistics were presented as median (range) or mean ± SD. The OS was calculated from the date of diagnosis of cancer to the date of death or the last follow-up. All patients were included in the intention to treat analysis. Survival was estimated as per the Kaplan-Meier method followed by log rank test. A value of *P* < .05 was considered statistically significant. The analysis was carried out using SPSS version 28, IBM Corp. Ltd, Armonk, NY, USA.

## Results

In patients who had undergone DEB-TACE before RT, partial response was observed in 9 patients and progressive disease in 1 patient after DEB-TACE. The patient treated with MWA had complete response. The patient treated with SIRT had partial response. Of the 2 patients who had received 7 doses of nivolumab, one had normalization of both serum AFP and PIVKA-II, while the other one had normalization of PIVKA-II and 83.3% decline in AFP. Of the 24 patients, 3 could not complete the planned course of RT. During the RT course, 1 patient had raised serum bilirubin after 4 fractions and 1 had grade 3 ascites after 3 fractions because of which the remaining fractions could not be given. One out of the 3 patients who was treated with abdominal compression belt had developed obstructed umbilical hernia after the second fraction of RT. The patient underwent primary repair of umbilical hernia and did not review for further fractions of RT. The changes in liver function and blood parameters during RT for all patients are noted in Table 3. The changes in tumour markers during RT are noted in Table 4.

**Table 3.** Changes in blood and liver function parameters during radiation therapy.

Parameter at diagnosis and before RT	Number	Parameter during RT	Number
Total leucocyte count before RT (per microlitre)	Range 2900-14 300	Neutropenia	
≥4000	16	Grade 3	1
<4000	8	Lymphopenia	
		Grade 2	7
		Grade 3	12
		Grade 4	4
Platelet count at diagnosis and before RT (per microlitre)		Thrombocytopenia (per microlitre)	
150 000 and above	11	75 000–150 000	4
100 000-150 000	4	50 000-75 000	7
50 000-100 000	9	25 000-50 000	2
Serum bilirubin before RT (mg/dL)	Range 0.46-3.0	Serum bilirubin increased	
		Grade 0	10
		Grade 1	4
		Grade 2	7
		Grade 3	3
		Serum bilirubin increased	
		Indirect bilirubin higher	10
		Direct bilirubin higher	3
		Both indirect and direct bilirubin equally raised	1
Serum albumin before RT (g/dL)	Range 2.3-4.7	Serum albumin decreased	
≥3.5	9	Grade 0	18
<3.5	15	Grade 1	2
		Grade 2	4
Serum AST before RT		Serum AST	
Raised 1.1 to 5 times ULN	24	No elevation	7
		Grade 1 elevation	2
		Grade 2 elevation	3
		Decreased	12
Serum ALT before RT		Serum ALT	
<30	4	No elevation	8
Raised 1.1 to 5 times ULN	20	Grade 1 elevation	2
		Decreased	14
Serum ALP before RT		Serum ALP	
≤120	2	Within normal limits	9
1 to 3 times ULN	20	Decreased	15
5.9 times ULN	1		
17.7 times ULN	1		
Serum GGT before RT		Serum GGT	
≤55	3	Within normal limits	11
1.6–5 times ULN	19	Decreased	13
7 times ULN	1		
10.4 times ULN	1		

Abbreviations: RT = radiation therapy; ULN = upper limit of normal.

Normal range: Platelet count = 150 000-450 000 per microlitre, serum albumin = 3.5-5.2 g/dL, serum total bilirubin = 0.2-1.1 mg/dL, serum direct bilirubin = 0.11-0.42 mg/dL, serum indirect bilirubin = 0.2-0.8 mg/dL, serum aspartate transaminase (AST) = 5-40 IU/L, serum alanine transaminase (ALT) = ≤30 IU/L, serum alkaline phosphatase (ALP) = 30-120 IU/L, and serum gamma glutamyl transpeptidase = ≤55 IU/L.

**Table 4.** Changes in tumour markers after radiotherapy and systemic therapy among the available patients for assessment at different time points.

Tumour marker	Before RT	After RT	At 4-6 weeks after RT	At 12 weeks after RT	At 24 weeks after RT
Serum AFP (ng/mL)	Median 357.9 Range 2.6-60488.6	Decreased	7	5	5
		Increased	9	7	2
		Same or normal	1	1	1
Serum PIVKA-II (mAU/mL)	Median 1301.9 Range 6-95194	Decreased	8	8	7
		Increased	7	3	1
		Same or normal	1	1	1

Abbreviations: AFP = alfa-fetoprotein; PIVKA-II = protein induced by vitamin K absence-II; RT = radiation therapy.

Normal range: serum alfafetoprotein = 0-8.5 ng/mL, serum PIVKA-II = 1-40 mAU/mL.

### Response and survival

**Figure 1.**
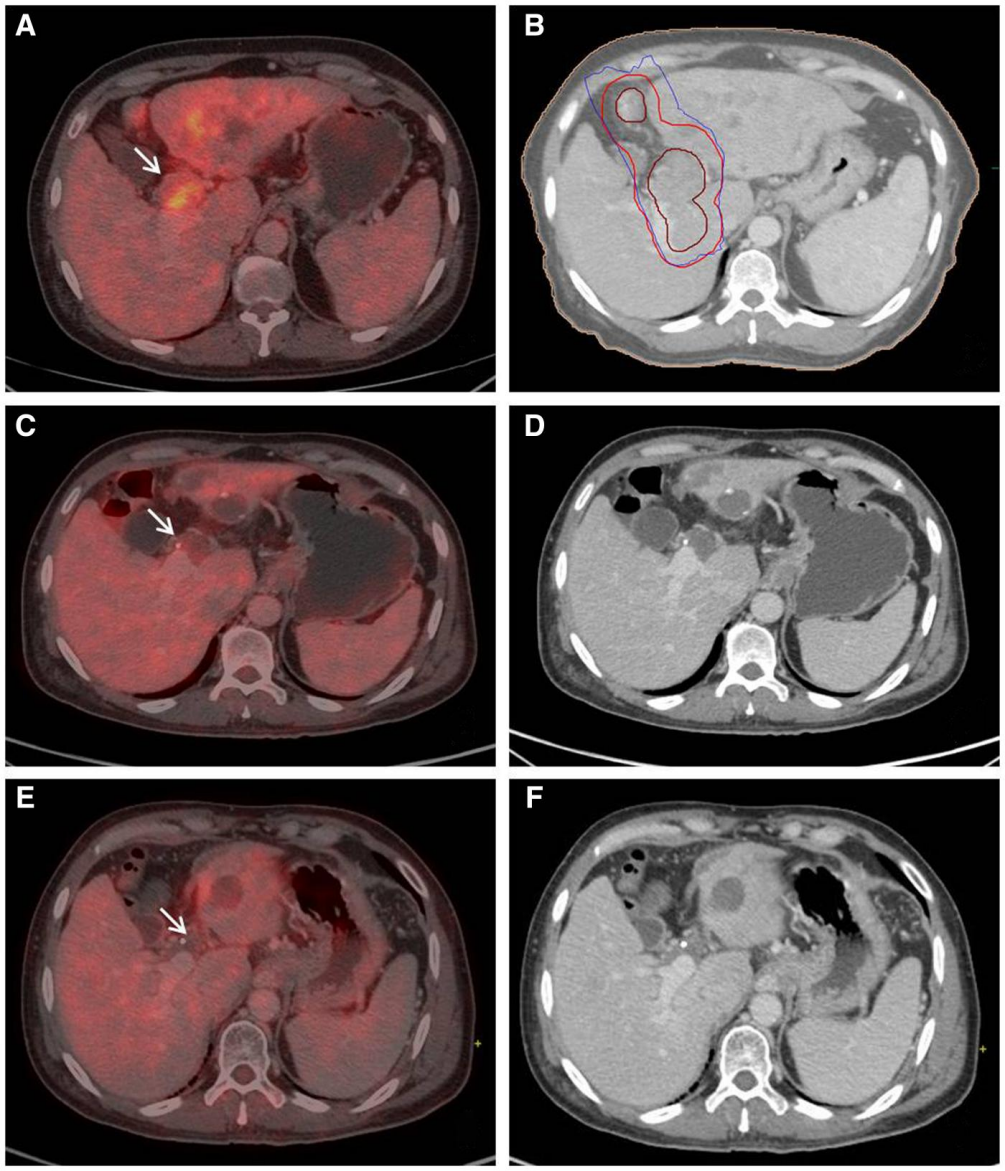
(A) Axial image of fused 18F-FDG PET-CECT shows FDG avid portal vein tumour thrombus (PVTT) (arrow) with lesion in left lobe; (B) Axial image of radiotherapy planning CECT on venous phase shows gross tumour volume delineation of PVTT along with planning target volume covered with 100% isoline; (C) and (D) Axial images of fused 18F-FDG PET-CECT and venous phase of CECT at 3 months after radiotherapy, systemic treatment, and DEB-TACE show FDG resolution and shrinkage of PVTT (arrow) with post DEB-TACE changes in left lobe lesion; (E) and (F) Axial images of fused 18F-FDG PET-CECT and venous phase of CECT at 1 year after radiotherapy with systemic targeted therapy and DEB-TACE shows complete response. Abbreviations: 18F FDG PET-CECT = 18Fluorine fluorodeoxyglucose PET–contrast-enhanced CT; CECT = contrast-enhanced CT; DEB-TACE = drug eluting beads transarterial chemoembolization.

**Figure 2.**
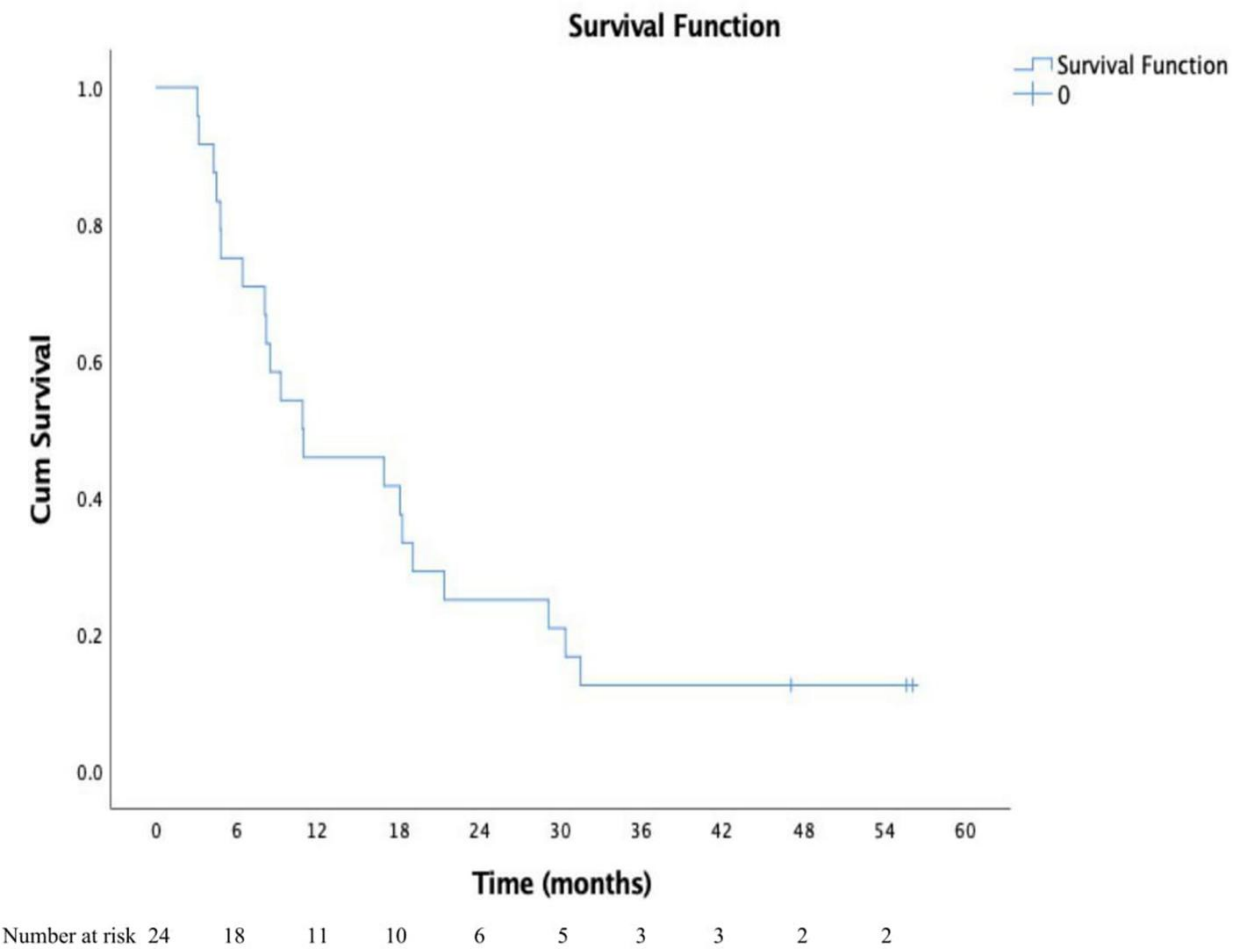
Kaplan-Meier graph shows overall survival from diagnosis of patients treated for hepatocellular carcinoma with portal vein tumour thrombosis.

Among the assessable patients with imaging available at 12 weeks after RT, in target, CR was noted in 1, PR in 7, SD in 4, and progressive disease (PD) in 1 patient. Three patients had developed new lesions in liver at 3 months. Considering the response in non-target lesions and development of any new lesions, the overall response at 3 months was CR (*n* = 1), PR (*n* = 6), SD (*n* = 2), and PD (*n* = 4). Among the assessable patients at 6 months after RT, in target, CR was noted in 4, PR in 2, and SD in 3 patients. The overall response at 6 months was CR (*n* = 4), PR (*n* = 2), SD (*n* = 2), and PD (*n* = 1). The patients showed CR or PR in terms of decreased enhancement, and FDG uptake with or without decreased extent and calibre in the PVTT. The treatment and follow-up imaging of 1 patient treated for HCC with PVTT with RT 30 Gy in 6 fractions to PVTT, sorafenib, DEB-TACE to parenchymal lesion, and further sorafenib followed by lenvatinib are illustrated in Figure 1. The ORR and DCR were 33.3% and 50% in the target (PVTT ± parenchymal tumour) and 29.2% and 37.5% in overall disease at 3 months. The ORR and DCR for overall disease were 25% and 33.3% at 6 months. The median follow-up for all patients was 10.9 months (range, 3.09-56.1 months). The median follow-up for the surviving patients (*n* = 3) was 55.6 months (47.1-56.1 months). The median OS was 10.9 months (95% CI, 0.74-21) for all patients. The 6-month, 1-year, 2-year, and 3-year survival rates were 75%, 45.8%, 25%, and 12.5% respectively (Figure 2). The response in PVTT (CR/PR versus SD/PD) at 3 months was significantly associated with OS (log-rank *P* = .036). The median OS was 30.4 months (95% CI, 12.1-48.7) versus 18.1 months (0.00-38.8) with CR/PR versus SD/PD. The 6-month, 1-year, and 2-year OS rates were 87.5%, 75%, 62.5% versus 80%, 60%, and 20% with CR/PR versus SD/PD, respectively.

**Figure 3.**
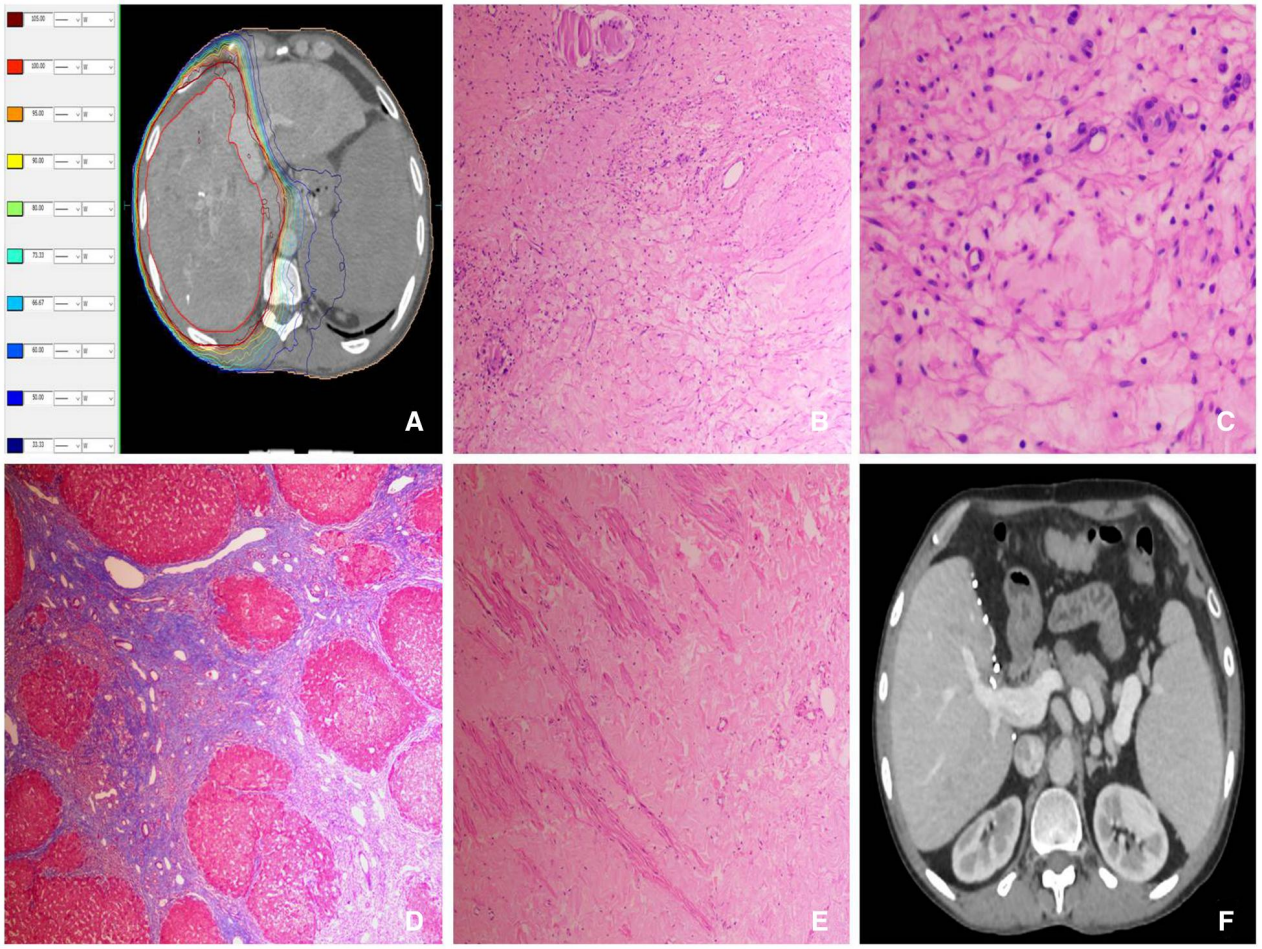
(A) Axial image of radiotherapy planning CECT on late arterial phase after DEB-TACE and targeted therapy shows gross tumour volume with isolines; (B) and (C) Photomicrograph shows tumour area in explant replaced by hyalinised tissue along with inflammatory cells (Haematoxylin and Eosin, 100× and 400×); (D) Photomicrograph shows cirrhosis in adjacent hepatic parenchyma of explant (Masson’s Trichome, 100×); (E) Photomicrograph shows portal vein thrombus in explant with wisps of smooth muscle fibres and part of hyalinised material with no malignant cells (Haematoxylin and Eosin, 100×); (F) Axial CECT image after surgery following multimodality treatment shows transplanted liver. Abbreviation: CECT = contrast-enhanced CT.

One 33-year-old patient diagnosed in 2019 with HCC with infiltrative lesion in right lobe measuring 15.7 × 10.8 × 15.9 cm with vp3 PVTT in RPV and branches had serum PIVKA-II of 12335.2 mAU/mL and normal serum AFP before RT. The patient had associated HBV related CLD which was diagnosed in 2011. The patient was treated with 2 sessions of DEB-TACE, RT, and sorafenib. The patient had decreased serum PIVKA-II at 6 and 12 weeks followed by normal level at 6 months after RT. After intolerance to sorafenib and in view of advanced disease at diagnosis, further systemic therapy was given with nivolumab along with lenvatinib. After completing 17 doses of nivolumab given every 2 weeks along with lenvatinib, the patient was maintained on lenvatinib alone. The patient was listed for liver transplant at 2 years from the diagnosis and underwent live donor liver transplantation (LDLT) at 3.5 years from the cancer diagnosis on November 4, 2022. The donor’s right portal vein from right lobe graft was anastomosed with main portal vein of the recipient. The interval between the last dose of nivolumab based immunotherapy and LDLT was 2 years. On gross pathological examination, the explant weighing 754 g and measuring 18 × 11 × 12 cm was observed. A necrotic lesion measuring 1.5 × 1.3 × 1.3 cm was seen in the right lobe. On microscopic examination, the lesion showed islands of hyalinization and coagulative necrosis. A part of a vessel showed myxoid degeneration and fibrin rich thrombus with no malignant cells. A complete post-treatment necrosis with no viable tumour was observed in the explant parenchyma as well as the portal vein. The adjacent liver parenchyma showed distortion of acinar architecture by bridging fibrous septae enclosing nodules of regenerative hepatocytes suggestive of cirrhosis. The portal tracts and septae showed mild to moderate lymphomononuclear cell infiltration. The hepatocytes showed mild macrovesicular steatosis of both large and small droplet type. The patient’s imaging before and after LDLT and pathological examination sections of explant are illustrated in Figure 3. The patient had developed clinical features of ischemic hepatitis at 3 months after LDLT and had recovered after management with intravenous steroid medication, antibiotics, human albumin infusion, nutritional, and other supportive medications. The patient is currently cancer- and liver disease-free at 1 year after LDLT and is on immunosuppressive protocol and has completed a follow-up of 55.6 months from the cancer diagnosis.

Another patient who was treated with a combination of nivolumab and RT is cancer-free and is alive with CLD at 47.1 months from diagnosis and on lenvatinib treatment with current age of 75 years. One patient is alive with intrahepatic parenchymal disease with CR in PVTT at a follow-up of 56.1 months and was counselled for liver transplant, however has opted for continuation of lenvatinib treatment. Of 24 patients, 2 had curative conversion with cancer-free status. Twenty-one patients had died of derangement in liver function with associated cancer. Distant metastases on follow-up were noted in lung (*n* = 1), spleen (*n* = 1), lung and bone (*n* = 1).

### Toxicity

Acute morbidity was grade 1-2 fatigue, nausea, anorexia, vomiting, or abdominal pain during RT in 13 patients. Two patients did not review for further investigations after RT, one of whom was advised sorafenib. Out of the 22 patients, 11 had derangement of liver function with deterioration in CP score of 2 or more points with raised bilirubin, reduced albumin, and/or ascites within 3 months after RT. Three out of the 11 patients could not receive systemic therapy after RT because of the decline in liver function. Three patients had deterioration in CP score by 1 point. One patient had a deterioration of CP score by 2 points because of intrahepatic tumour progression. Seven patients did not have a change in CP score within 3 months after RT. Of the 7 patients, 1 did not receive systemic therapy. One patient treated with RT to PVTT, sorafenib, and DEB-TACE had a late toxicity with gastrointestinal bleeding with antral vascular ectasia on endoscopy at 5 months after RT. The patient was treated with argon plasma coagulation and low dose oral steroids after which the bleeding was controlled. The patient is currently on lenvatinib and is alive with cancer and CLD at 56.1 months from the cancer diagnosis.

## Discussion

The present study showed the benefit of radiotherapy with systemic therapy and DEB-TACE in patients with HCC with PVTT. With modern technologies and image guidance, precision radiation offers favourable outcomes. Hypofractionated stereotactic radiotherapy uses fewer fractions of higher dose as compared to the conventionally fractionated radiotherapy. A statistically better median survival of 13.3 months with stereotactic radiotherapy as compared with 9.8 months with fractionated radiotherapy has been reported for HCC with PVTT.^9^ Furthermore, stereotactic radiotherapy requires a shorter overall treatment time allowing early initiation of systemic treatment to prevent intrahepatic and/or distant progression of disease. Stereotactic radiotherapy has been reported to be a more time-efficient treatment as compared with IMRT.^10^ The dose constraints for different fractionation regimens of definitive radiotherapy for HCC with PVTT have been reported in a study.^11^ In order to improve the overall disease control with multimodality treatments while maintaining liver function, a relatively lower dose per fraction with the stereotactic technique was used in the present study.

The combination of lenvatinib with RT has shown to result in a significantly better survival as compared to lenvatinib alone in a retrospective study of patients with HCC with PVTT.^12^ In the study, lenvatinib was initiated within 1 week after the last fraction of RT. The median survival with the combination treatment as compared with the single treatment was reported as 19.3 versus 11.2 months, respectively. No increase in adverse events was observed with the combined treatment. Pharmacokinetic studies have revealed synergistic effect of the combination of radiotherapy and sorafenib.^13^ The combination of RT with sorafenib compared with sorafenib was devised in the RTOG 1112 protocol in the patients with HCC.^14^ The synergistic effects of the combined treatment with RT and immune checkpoint inhibitors have been highlighted in the emerging scientific evidence.^15^^,^^16^ In a retrospective analysis of 80 patients with HCC with PVTT and treated with RT, a median survival of 11.5 months was observed.^17^ The 6- and 12-month survival rates were 74.5% and 47.5%, respectively. A similar 6- and 12-month survival rates of 75% and 45.8% were observed in the present study. In a recently published study on combination treatment in HCC with PVTT, RT with a single dose of 6-8 Gy was given within the first week of starting tyrosine kinase inhibitor and immunotherapy.^3^

The addition of locoregional treatments to systemic therapy in patients with PVTT has shown an impact on survival of the patients offering a chance of possible cure with surgery.^18^ The combined locoregional treatment with RT and TACE has been evaluated in patients with inoperable HCC with PVTT in a meta-analysis.^19^ With a RT-TACE interval preferably <28 days, better ORRs and OS rates have been observed with the combination therapy as compared with monotherapy. In patients with type III PVTT, a combination of TACE and radiotherapy has shown the most beneficial outcome as compared to TACE, TACE with sorafenib, or surgical treatment.^2^ In the present study, TACE was combined with RT as per the patients’ clinical suitability. With the standard therapies, a significant proportion of patients with liver cancer present with pain at later stages of progression which can be treated with radiotherapy as reported in a phase 3 study.^20^ The patients with HCC (*n* = 23) or liver metastases (*n* = 43) unsuitable or refractory to standard therapies were included in the study. A significant improvement in hepatic pain with whole or near whole liver single fraction 8 Gy palliative radiotherapy was reported as compared with best supportive care.^20^

The FDG uptake of PVTT Is an important consideration for deciding on LDLT for patients. A significantly better survival has been reported in PVTT responders as compared with non-responders.^21^ The PVTT to liver uptake ratio was a significant factor associated with OS and PFS on multivariate analysis in a study of 166 patients from Korea.^22^ The median survival was observed at 10.1 months in patients with no extrahepatic metastases. After neoadjuvant radiotherapy with hepatic arterial infusion chemotherapy with 5-fluorouracil and cisplatin in patients with HCC with PVTT undergoing LDLT, no viable tumour thrombi in major vessels have been reported.^23^ One of the patients from the present study managed with multimodality treatment showed a favourable outcome with post-therapy complete necrosis in liver parenchyma as well as portal vein on the explant specimen. Similar complete pathological responses with necrosis have been reported after pre-operative RT for PVTT.^24^

The incorporation of radiation in the form of endovascular brachytherapy with stenting in combination with TACE and systemic therapy have demonstrated improved survival rates.^25^^,^^26^ The addition of 125Iodine seed brachytherapy with TACE, lenvatinib, and programmed death-1 inhibitor has shown to result in an improved disease control rate and median survival of 21 months (95% CI, 18.4-23.5) in HCC with PVTT.^26^ The median OS rates without brachytherapy were 14 months (95% CI, 10.7-17.2) and 10 months (95% CI, 7.8-12.1) in patients treated with TACE, lenvatinib, and PD-1 inhibitor and TACE with lenvatinib, respectively.

The dose–response relationship for liver tumours has been reported based on the authors’ experience from different centres.^27^ A study reviewed data of patients with HCC treated with median dose of 4 Gy per fraction (range 2-10 Gy).^28^ A 50% local control at 6 months was demonstrated with 2 Gy per fraction equivalent dose (EQD_2_) of 53 Gy versus 70 Gy with α/β = 10 Gy for HCC versus metastatic colorectal liver tumours.^28^ The planning techniques were employed so as to maintain PTV maximum point dose to 107% and PTV mean dose was similar to the prescribed dose. The median tumour diameter was 6.6 cm and 5 cm for HCC and metastatic tumour groups, respectively. The study demonstrated relative radiosensitivity of HCC as compared with other tumours in liver. No clear evidence for dose–response relationship has been observed for RT of HCC and optimal local control rates have been seen with even low-dose conservative RT regimens.^29^ In addition, the other local, regional, and/or systemic treatments with radiotherapy lower the viable tumour load. Among the various disease-related factors, the tumour size impacts the local control after radiotherapy.^29^ Furthermore, there is heterogeneity in dose prescription coverage to PTV in the literature which makes the pooling of outcomes from different studies challenging.

Non-classic radiation induced liver disease (RILD) has been defined as elevated transaminases more than 5 times upper limit of normal or CTCAE grade 4 in patients with baseline more than 5 times upper limit of normal, or decline of liver function with worsening of CP score by 2 or more within 3 months after the completion of radiotherapy.^30^ In the present study, the deterioration of liver function was a combined effect of RT and systemic therapy and was also influenced by the additional liver-directed treatments. It is pertinent to note that the radiation dose fractionation for HCC with PVTT needs to be balanced with the liver function in order to enable timely initiation and continuation of systemic therapy.

The limitations of the present study were that it included patients with varying extent of intrahepatic disease. The disease extent that was covered in the radiation target volume and the systemic therapy drug varied as well among patients. However, the study underlined the potential role of various therapeutic combinations in the management of HCC with PVTT.

The present study showed the benefit of radiotherapy with systemic therapy and DEB-TACE in reducing tumour burden and decreasing the risk of locoregional and/or distant progression in HCC with PVTT. Radiotherapy as part of the multimodality treatment offers the potential to improve disease control and survival in patients with HCC with PVTT. The combination of radiotherapy with different systemic therapy drugs needs to be evaluated in further studies to allow individualised treatment for patients with hepatocellular carcinoma with PVTT.

## Data Availability

The data analysed for the study is available within the article.
